# Maternal dietary glycaemic change during gestation influences insulin-related gene methylation in the placental tissue: a genome-wide methylation analysis

**DOI:** 10.1186/s12263-019-0634-x

**Published:** 2019-05-09

**Authors:** Weili Yan, Yi Zhang, Liping Wang, Wenhong Yang, Chunying Li, Liling Wang, Ping Gu, Yingqian Xia, Juhua Yan, Ying Shen, Qian Zhao, Dayan Niu, Kai Mu, Yuan Jiang

**Affiliations:** 1 Department of Clinical Epidemiology, Children’s Hospital of Fudan University, 399 Wanyuan Road, Shanghai, 201102 China; 2 Maternity and Child Health Center, International Peace Maternity & Child Health Hospital of China Welfare Institute, 910 Hengshan Road, Shanghai, 200030 China; 3 Department of Genetic Counseling, Kunshan Maternity and Child Care Center, 458 Western Tongfeng Road, Kunshan, 215300 China

**Keywords:** Pregnant women, Glycaemic index, Insulin, Placenta, DNA methylation, Epigenome

## Abstract

**Background:**

Studies have shown that the effects of maternal nutrition exposure during gestation influence metabolic risk in early life through an epigenetic mechanism. Low glycaemic index (GI) diets benefit both maternal and neonatal gestational outcomes. We hypothesize that maternal dietary GI or glycaemic load (GL) changes during pregnancy impact placental DNA methylation, especially in insulin resistance-related genes.

**Methods:**

From a clinical trial of overweight pregnant women, 12 subjects who successfully reduced their GI and another 12 whose GI increased despite the intervention were selected. A genome-wide differential methylation analysis of placental tissue DNA was conducted, followed by bioinformatic annotation and validation analysis. The distribution of genome-wide differentially methylated regions (DMRs) and CpG sites was described. Six CpG sites in regulatory regions of four insulin-related genes (*PLIN1*, *CPT1B*, *SSTR4*, and *CIDEA*) were selectively validated by pyrosequencing. Pairwise Spearman correlation analysis was performed to test methylation–phenotype association in an additional 153 subjects from the same trial. Correlation between methylation of significant sites and placental mRNA expression of *SSTR4* was also analysed.

**Results:**

Dietary GI decreased by 24.3 (26.2–20.1) in the group who responded appropriately to the intervention and increased by 19.6 (15.2–29.1) in the comparison group. Epigenome-wide analysis identified 108 DMRs and 365 CpG sites with *P* < 0.05 adjusted by false discovery rate, distributed over all chromosomes. The methylation level of cg05009389 in the 3′ UTR of *PLIN1* was negatively correlated with maternal weight gain (*ρ* = − 0.21, *P* = 0.027) and increase in insulin levels (*ρ* = − 0.24, *P* = 0.015) during gestation. Methylation levels of cg17586860 and cg18197392 in the 5′ UTR region of *SSTR4* were negatively correlated with changes in dietary carbohydrate intake (*ρ* = − 0.24, *P*s ≤ 0.006) and GL across gestation (*ρ* = − 0.23, *P*s ≤ .008). This correlation survived the adjustment for maternal factors such as dietary GI, body mass index, and gestational diabetes. Up to 89% of cg18197392 methylation was explained by GL change. Cg14631053 methylation correlated positively with mRNA expression of *SSTR4* in the placenta (*ρ* = 0.20, *P* = 0.037)*.*

**Conclusions:**

We provide the first evidence that maternal dietary GI changes during gestation may impact placental DNA methylation of insulin regulation genes. This supports the hypothesis that placental methylation may be the epigenetic mechanism through which maternal diet influences the metabolic health of offspring.

**Electronic supplementary material:**

The online version of this article (10.1186/s12263-019-0634-x) contains supplementary material, which is available to authorized users.

## Background

Maternal obesity or excessive weight gain during pregnancy is recognized as an important issue with a globally increasing trend [1, 2]. Excessive weight gain during pregnancy is associated with multiple maternal and neonatal complications and may have long-term unfavourable effects on the long-term risk of developing metabolic disorders in offspring [3]. A low glycaemic index (GI) diet, as a dietary intervention for pregnant women with high metabolic risk, has been successfully shown to improve maternal gestational outcomes [4, 5], reduce the need for insulin among women with gestational diabetes [6], and reduce neonatal birth weight [7] and incidence of births that are clinically considered “large for gestational age” [4]. Epigenetic processes are suggested to be an important link between maternal gestational dietary exposures and altered offspring metabolism in animal studies [8, 9]. In recent years, emerging evidence from human studies suggests that various types of maternal in utero exposure, such as cigarette smoking [10–12], gestational diabetes [13, 14], insufficient maternal weight gain [15], and even maternal anxiety [16], can influence methylation in the placental tissue or cord blood. Some gene methylation changes in these tissues related to maternal gestational diabetes are also linked with adiposity-related outcomes in childhood [17]. A recent study reported that maternal glycaemia during pregnancy is correlated with placental DNA methylation levels of brown adipose tissue-related genes, and variations in DNA methylation levels of these same genes are correlated with cord blood leptin levels [18]. Another study reported that higher retinoid X receptor alpha *(RXRA*) gene promoter methylation levels in the umbilical cord tissue were associated with lower maternal carbohydrate intake in early pregnancy and child adiposity at 9 years of age [19]. However, evidence is lacking as to whether maternal GI diet changes during gestation are associated with offspring gene methylation changes.

## Results

Clinical characteristics of the two groups of study samples for genome-wide analysis and validation analysis are shown in Table 1. In terms of the discovery study sample for genome-wide differential methylation analysis, the GI decreased from 67.6 to 42.8 with a change of 24.3 (26.2–20.1) in the decreased GI group and increased from 51.2 to 73.5 with a change of 19.6 (15.2–29.1) in the increased GI group across the gestational period. There were no additional significant differences in the other characteristics at baseline or in pregnancy outcomes at delivery between the two groups. In the validation study sample, the GI decreased 1.1 on average but with larger variability. The GI increased in 76.0% of subjects and decreased in 24.0% of subjects (data were not shown).

**Table 1:** Characteristics of the study populations

	Discovery sample (*n*=24)			Replication sample (*n*=153)
	Case group (*n*=12) Diet GI decrease	Control group (*n*=12) Diet GI increase	*P*	
Maternal characteristics				
Diet GI				
Baseline	67.6(60.9, 73.9)	51.2(38.5, 57.8)	<0.001	64.0±10.3
Middle gestation	68.7(56.7, 71.8)	66.0(59.0, 68.1)	0.538	62.1±10.9
Late gestation	42.8(38.9, 47.6)	73.5(60.9, 76.2)	<0.001	62.8±10.7
Change of GI^a^	-24.3(-26.2,-20.1)	19.6(15.2, 29.1)	<0.001	-1.1±13.2
Change of GL^a^	-68.8(-114.4, -16.1)	12.8(-55.3, 64.4)	0.024	-11.1(-57.1,36.2)
First antenatal visit				
Maternal age, year	30.2±3.0	30.9±4.5	0.638	29.4±3.7
Gestational week, week	12.9±1.2	12.4±1.5	0.378	12.6±1.7
Height, cm	164.3±5.9	163.8±5.8	0.850	163.1±6.1
Weight, kg	78.6±10.5	76.3±9.9	0.584	76.0±8.7
BMI, kg/m^2^	29.1±3.0	28.4±3.3	0.624	28.5±2.8
SBP, mmHg	122.9±6.7	126.8±19.7	0.521	122.4±14.7
DBP, mmHg	78.8±6.6	76.2±10.4	0.477	73.9±13.3
FPG, mmol/L	4.7±0.4	4.7±0.6	0.952	4.6±0.5
Insulin, uU/mL	8.3(5.7, 16.5)	7.8(6.7,12.0)	0.768	8.6(5.8,13.6)
HOMA-IR	1.8(1.1,3.5)	1.6(1.2,2.3)	0.573	1.9(1.1,2.9)
HbAlc, %	5.2±0.3	5.5±0.4	0.129	5.3±0.4
Triglyceride, mmol/L	1.9±1.0	2.2±1.0	0.500	1.7±0.8
Total cholesterol, mmol/L	4.9±0.8	4.6±0.7	0.307	4.8±0.7
Maternal outcomes				
Gestational week, week	39.9±0.9	39.5±1.5	0.510	39.8±1.4
Weight, kg	89.4±11.8	85.2±10.8	0.380	86.0±10.1
GWG, kg	10.8±3.5	9.0±8.3	0.492	10.1±5.2
Insulin, uU/mL	8.9(7.1,9.0)	11.5(8.5,11.7)	0.566	10.3(6.7,13.1)
HbAlc, %	5.1±0.5	5.4±0.6	0.400	5.3±0.4
GDM, n (%)	4(33.3)	6(50.0)	0.408	33(31.4)
Gestational hypertension, n (%)	4(33.3)	2(16.7)	0.640	24(21.2)
Neonatal outcomes				
Cord blood C peptide, ng/mL	0.7±0.4	0.9±0.5	0.289	0.9±0.8
Birth weight, g	3754.6±597.6	3327.1±456.8	0.062	3476.1±541.5
Low birth weight, n (%)	0(0)	1(8.3)	1.000	3(2.7)
Macrosomia, n (%)	3(25.0)	2(16.7)	0.615	15(13.3)
Length, cm	50.4±0.9	49.8±1.1	0.110	50.1±1.0
Preterm, n (%)	0(0)	1(8.3)	1.000	3(2.7)

*GI* glycemic index, *GL* glycemic load, *BMI* body mass index, *SBP* systolic blood pressure, *DBP* diastolic blood pressure, *FPG* fasting plasma glucose, *HOMA-IR* homeostasis model assessment of insulin resistance, *GWG* gestational weight gain, *GDM* gestational diabetes mellitus

^a^Change of GI and GL were calculated as values at baseline minus values at late gestation

**Fig. 1:**
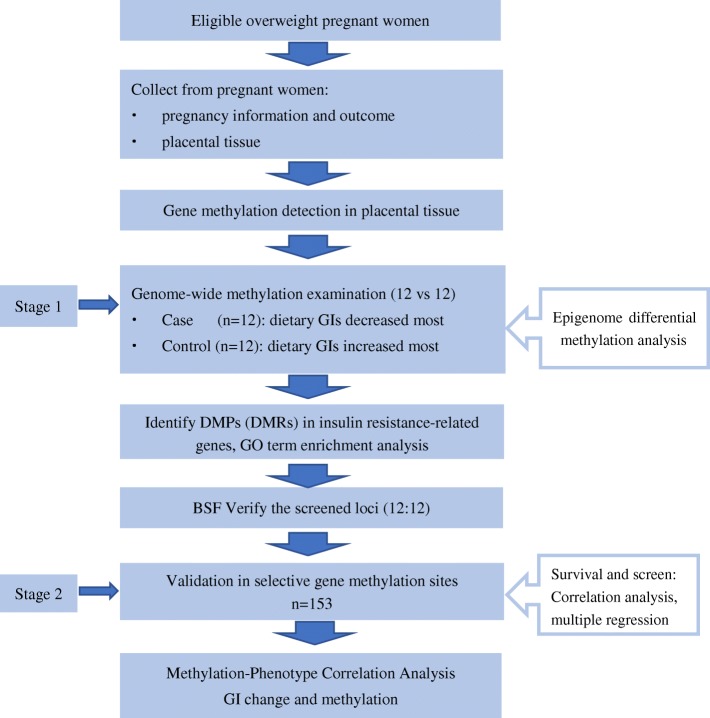
The flow chart of the current study

The genome-wide differential methylation analysis (step 1) identified 108 DMRs with adjusted *P* < 0.05 (raw DMR *P* ranged from 10^−24^ to 10^−3^), including 365 CpG sites, and mapped to 95 genes (details in Additional file 1). These CpG sites were distributed across all autosomal chromosomes, with all possible relationships to CpG islands, including within CpG islands, the open sea, the shelf, or the shore of CpG islands (Fig. 1). Among these sites, 149 (40.8%) sites were hypomethylated and 216 (59.2%) sites were hypermethylated. After FDR adjustment, 18 enriched GO terms in the interaction network were genomically significant (FDR < 0.05) (Additional file 2) (step 2). The most significant terms referred to biological processes, such as positive regulation of signalling.

From 365 differentially methylated sites, those in regulatory regions of insulin regulation genes indicated by GO term analysis were selected for validation analysis (step 3). Ten CpG sites in *PLIN1*, *CPT1B*, *SSTR4*, and *CIDEA* that were validated were hypomethylated in the decreased GI group (Table 2). Among which, four sites failed for technical reasons and the remaining six sites (all located in CpG islands) were examined by pyrosequencing in the validation cohort and were included in further methylation–phenotype correlation analysis, one for *PLIN1*, *CPT1B*, and *CIDEA* and three for *SSTR4.* The pairwise methylation–phenotype analyses revealed several weak correlations (Table 3): cg05009389 in the 3′ UTR of *PLIN1* was negatively correlated with maternal gestational weight gain (*ρ* = − 0.21, *P* = 0.027) and an increase of insulin across gestation (*ρ* = − 0.24, *P* = 0.015), cg17586860 and cg18197392 in the 5′ UTR region of *SSTR4* (transcription starting site, TSS200) were negatively correlated with the change in carbohydrate intake (*ρ* = − 0.24, *P*s ≥ 0.006), and GL changes across the gestation period (*ρ* = − 0.23, *P*s ≤ 0.008). We did not find significant correlations of cg14631057 methylation with any clinical phenotypes; however, it showed moderate correlations with the methylation of the other two sites in the same CpG island in the promoter of *SSTR4* (*ρ* was 0.53–0.59, *P*s < 0.0001). As shown in Table 4, further multivariate regression analyses showed that the GI at the first interview and GI change during gestation, as well as BMI and gestational diabetes mellitus (GDM), explained 9.3% of the total variance of cg17586860 methylation. The GI change was independently associated with cg17586860 methylation levels (*β* = − 0.36, *P* = 0.003), explaining 72.8% of the variance after adjustment for covariates. No significant results were found for the other CpG sites (Table 4).

**Table 2:** Methylation variable positions located in regulatory region of *PLIN1, CPT1B, SSTR4, and CIDEA gene*

CpG	Closest gene	Regulation region	CpG island	Methylation difference	*P*	FDR
cg26585724	*PLIN1*	3'UTR	shore	-0.116	0.0007	0.040
cg05009389	*PLIN1*	3'UTR	island	-0.206	0.0007	0.022
cg10490842	*CPT1B*	5'UTR	island	-0.031	0.0003	0.041
cg19112186	*CPT1B*	5'UTR	island	-0.038	0.0003	0.030
cg05156901	*CPT1B*	5'UTR	island	-0.042	0.0003	0.025
cg10770023	*CPT1B*	5'UTR	island	-0.052	0.0003	0.035
cg14631053	*SSTR4*	TSS200	island	-0.066	0.0001	0.006
cg17586860	*SSTR4*	TSS200	island	-0.075	0.0001	0.009
cg18197392	*SSTR4*	TSS200	island	-0.099	0.0001	0.020
cg20950011	*CIDEA*	TSS1500	island	-0.049	<0.0001	0.043

Methylation difference was calculated as levels in the GI decrease group minus that in the GI increase group

**Table 3:** Correlations between phenotypes and placental DNA methylation

CpG	Gene	Weight gain	Ch_insulin	Ch_Carbs	Ch_Fiber	Ch_GL	Ch_GI
cg05009389	*PLIN1*	-0.20*	-0.21*	-0.01	0.18	0.01	-0.18
cg10490842	*CPT1B*	-0.14	0.02	-0.08	-0.18	-0.06	-0.06
cg14631053	*SSTR4*	-0.02	0.08	-0.14	0.03	-0.10	0.01
cg17586860	*SSTR4*	-0.01	0.06	-0.24*	-0.04	-0.23*	-0.08
cg18197392	*SSTR4*	0.02	0.15	-0.24*	-0.08	-0.23*	-0.02
cg20950011	*CIDEA*	-0.02	-0.02	-0.10	-0.04	-0.05	0.01

Weight gain was defined as difference of body weight before delivery minus-body weight at first antenatal visit; Ch_ of diet intakes, levels at endpoint minus levels at baseline

Values wereρ(correlation coefficient from Spearman correlation analysis); *P<0.05

**Table 4:** Association between dietary GI change across gestation and placental methylation levels of CpG sites based on stepwise regression analysis

Dependent variables	F_model_	P_model_	Adjusted Model R^2^, %	Dietary GI change			Significant covariates
				P	β(95%CI)	EV,%	
cg17586860	4.33	0.007	9.3	0.003	-0.36	72.8%	GDM, GI-baseline
cg20950011	3.04	0.053	4.0	>0.05	-	-	GDM, BMI

Only significant models were presented. Maternal age, BMI, HOMA-IR and dietary GI at baseline, GDM, gestational age at delivery and gestational weight gain were adjusted as covariates

*EV* explained variation

Among the three CpG sites in the CpG island of the *SSTR4* promoter, the cg14631053 methylation levels were correlated with the placental mRNA expression of *SSTR4* (*ρ* = 0.20, *P* = 0.037), and no significant correlations were found for the other two CpG sites.

## Discussion

By using genome-wide DNA differential methylation analyses followed by validation analyses, we provide evidence suggesting that maternal dietary glycaemic changes during gestation are associated with placental DNA methylation changes in insulin resistance-related genes. We identified 108 DMRs across the whole genome including 365 CpG sites and 95 genes. By using a larger sample size, we also found consistent weak correlations between methylation levels of CpG sites in the promoter region of four insulin resistance-related genes with maternal dietary glycaemic change and with changes of some other related clinical phenotypes across gestation: methylation of one CpG site in the 3′ UTR of *PLIN1* gene and two sites of a CpG island close to TSS of *SSTR4* gene are associated with maternal changes of dietary GI, GL, weight gain, and insulin levels during gestation. In addition, methylation of one CpG site from the same CpG island in *SSTR4* is weakly correlated with the placental mRNA expression of *SSTR4* gene. These results suggest that placental DNA methylation may be altered as a response to significant changes in maternal diet GI, even in a short period of gestation (approximately 20 weeks). The methylation and gene expression alterations in regulatory regions of insulin resistance-related genes in the placental tissue may be the link between maternal diet modifications with foetal outcomes or future metabolic risks, which is consistent with some previous clinical studies.

One of our findings is that maternal dietary glycaemic changes are associated with methylation alterations in hundreds of genes across the genome. In combination with previous studies, these findings support the epigenetic impact of maternal nutritional exposure during gestation on offspring metabolic risk. Some previous studies focused on the impact of maternal dietary protein and fat intake [8, 9, 20]. Godfrey et al. [21] reported associations of lower maternal carbohydrate intake in early pregnancy and hypermethylated RXRA genes in the umbilical cord tissue of healthy neonates and the association between this hypermethylation with children’s fat mass at age 9. In the current study, based on the placental tissue instead of the umbilical cord tissue, we did not find significantly differential methylation of the *RXRA* gene between pregnant women with distinct and opposite dietary glycaemic changes. Ruchat et al. reported that maternal GDM epigenetically affects genes predominantly involved in metabolic diseases; however, the placental tissue and cord blood share only 25% of differentially methylated CpG sites [22]. In our study, *PLIN1* and *SSTR4*, whose methylation patterns are associated with maternal dietary GI change and related phenotype change, are not in the gene list of the previous study [22]. Park et al. found that the obesity status of women influences gene methylation changes in response to folic acid supplementation [23]. Similarly, only some of the genes had similar methylation changes in the cord blood sample and placental samples. It is known that DNA methylation is widely dynamic and tissue-specific during the development of humans [22], the cellular composition of placentas varies among individuals [3], and the methylation profiles differ in different cell types in human placentas [24]. Although the collection of the placental tissue followed standardized procedure, the heterogeneity of sampling by different investigators may have led to heterogeneity in the cell type composition of the placental tissue and may have an impact on methylation levels of selected genes. However, this impact on methylation is likely to be non-directional, making it less likely to be a source of bias to the weak associations found in this study.

The *SSTR4* gene encodes the type 4 receptor of somatostatin that exerts inhibitory effects on all endocrine and exocrine secretions in humans, including its role as an endogenous inhibitor of cell proliferation [15] and function in certain areas of the central nervous system, such as motor, sensory, behavioural, cognitive, and autonomic effects [20]. The *SSTR4* gene is expressed in human placental tissue [21]. The CpG site, cg17586860, survived the two-stage association analysis and is correlated with maternal GL change and with methylation patterns of other sites (cg14631053 and cg18197392). This site is located in the TSS200 region of *SSTR4*, where sequences are with potentially important functions, such as binding sites of several transcription factors and elements required for tissue-specific promoter activity [25, 26]. Previous studies conducted transfection experiments focusing on this region but did not indicate any effects of possible regulators on transcriptional regulation of *SSTR4*. Sequence analysis of the *SSTR4* gene did not find the proximal 5′ UTR to contain any potential TATA or CAAT boxes but that it was highly GC-rich within the first 300 bps [26], which contains a CpG island. The correlation we find that the reduced methylation of cg17586860 in this island in relation to greater dietary GL decrease (weak negative correlation) may support the hypothesis that maternal dietary glycaemic change may have a favourable impact on *SSTR4* mRNA expression through the alteration of methylation status of promoter region and possible further effects on foetal development. Unfortunately, placental *SSTR4* protein expression was not examined in the current study. It still remains unclear how the gene methylation and mRNA expression modifications observed may affect the structure or the function of the placenta or other potential target organs since the evaluation of the proteins regulated by the genes studied is not available. Future study is expected to examine the effects of reduced expression of *SSTR4*, possibly including the enhancement of various endocrine and exocrine secretions in the placenta.

Another interesting finding is the differential methylation of cg05009389 in the 3′ UTR of the placenta *PLIN1* gene in relation with extreme maternal dietary GI change during pregnancy. We found weak correlations between reduced methylation with greater maternal insulin increase and body weight gain. The *PLIN1* gene encodes perilipin-1, which coats lipid storage droplets in adipocytes and functions as inhibitors of lipolysis [20]. Godfrey et al. indicated a gene–nutrient interaction between a *PLIN1* polymorphism and dietary intake of saturated fat and carbohydrates and their effect on insulin resistance levels [21]. Loss-of-function mutations in *PLIN1* have been reported to lead to familial partial lipodystrophy, severe insulin resistance, and diabetes [22]. *PLIN1* promoter methylation was observed to be inversely correlated with *PLIN1* mRNA expression and lipolytic activity [23]. This evidence indicates epigenetic regulation of the gene and its function. The findings of our study may support a possible epigenetic mechanism of the influence of maternal dietary glycaemic change on offspring’s future risk of lipid metabolic disorders. Unfortunately, *PLIN1* is commonly expressed in adipose tissue and not in human placenta tissue, so examining its expression was not possible in the current study.

Strengths of our study include, firstly, the use of human placental tissue to assess the epigenetic effects of maternal dietary GI change during pregnancy on DNA methylation in offspring. Secondly, the application of the Infinium 450K BeadChip technology to assess genome-wide methylation profiles offered greatly improved genomic coverage over the earlier 27K platform. Thirdly, in the current study, the maternal dietary glycaemic change was as a result from counselling, not from directly receiving low glycaemic food. Its associations with placental tissue gene methylation support the importance of maternal lifestyle modification during gestation.

This study has several limitations. Firstly, the subjects included in our study were from a randomized controlled trial and limited as to whose placental tissues were successfully collected and processed. Thus, the interpretation of associations identified in the current study sample should be taken with caution. Secondly, the sample size for KEGG pathway and GO analyses was relatively small. Thirdly, only the subgroup of CpG sites (in the regulatory region of genes that related to insulin resistance) was chosen from the genome-wide differential methylation analysis for methylation–phenotype correlation analyses in the larger study sample.

## Conclusions

We report for the first time that maternal dietary GI changes are associated with offspring’s placental DNA methylome, including in insulin resistance-related genes. Methylation in the promoter regions of *PLIN1* and *SSTR4* is correlated with maternal changes in dietary GI, GL, and other clinical phenotypes. This evidence supports the idea that placenta methylation alteration may be the epigenetic mechanism underlying favourable effects of maternal dietary habit changes on their offspring. Future work is needed to determine the relevance of epigenetic changes in human placenta tissue to the change of maternal GI during pregnancy.

## Methods

### Participants

**Fig. 2:**
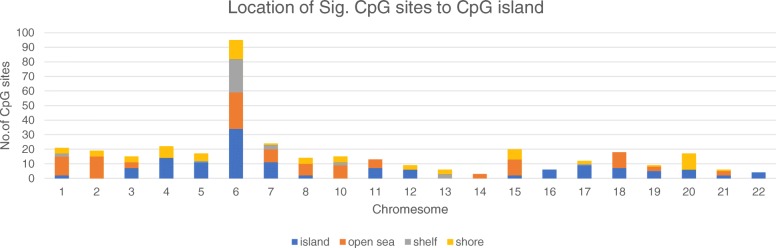
Distribution of differentially methylated CpG sites (raw *P* < 0.05) by chromosome and relationships to CpG islands (*n* = 365). Island, CpG island; open sea, 4 kb away from island; shelf, 2 kb away from island; shore, within 2 kb from island

Participants were selected from a lifestyle intervention study (clinicaltrials.gov, NCT01628835), which was carried out at two maternity and child care centres in southeast China from June 2012 to October 2015. In brief, overweight pregnant women were recruited at the first antenatal examination and received dietary counselling three times during gestation; the control group received standard dietary counselling according to the nationwide nutritional recommendations, and the experimental group additionally received an accurate assessment of their personal dietary glycaemic index and advice about low glycaemic diets, when needed. Overall, all mother–child pairs with completed data in terms of prenatal exposure (including their individual change in dietary GI on each visit) and outcomes at birth (including a collection of placental tissue samples) were included in the current study (*n* = 177). Of these, the 12 subjects whose GI decreased the most remarkably during gestation were chosen as the “GI decrease group,” and 12 subjects whose GI increased most remarkably, despite intervention to lower their GI, were chosen as the “GI increase group,” for genome-wide differential methylation analysis. The remaining 153 mother–child pairs were treated as a validation sample for methylation-phenotype association analysis (Fig. 2).

### Maternal dietary GI measurements during gestation

Maternal dietary data were collected by 24-h dietary recall at three face-to-face dietary consultation interviews. These interviews were incorporated as a part of the subjects’ routine antenatal care visits to improve compliance. The baseline interview was conducted at the first antenatal visit. The second interview was arranged to coincide with the conventional 75-g oral glucose tolerance test (OGTT) during the 24th to 28th week, and the third interview coincided with the usual liver function test during the 34th to 36th week. Dietary GL, GI, and nutrients were evaluated using a customized Excel worksheet that incorporated the Chinese [27] and international food composition tables and published GI values [28–30]. The change in GI during pregnancy was calculated as the GI at the third interview minus the GI at the first interview.

### Data and biological sample collection

Demographic, clinical, and anthropometric data were collected on the first visit (baseline). Body mass index (BMI) was calculated as weight (kg)/[height (m)]^2^ and categorized according to Chinese categories (underweight < 18.5 kg/m^2^, normal weight 18.5–23.9 kg/m^2^, overweight 24.0–27.9 kg/m^2^, and obese ≥ 28 kg/m^2^) [31]. The gestational age was estimated from the last self-reported menstrual period and corrected by the first routine ultrasound examinations around the 16th week of gestation. Routine lab examination data, including plasma glucose, serum insulin, and glycosylated haemoglobin, were obtained from the hospital information system. The insulin resistance index was calculated (under a model of homeostasis) using the following formula [32]: [fasting plasma glucose (mmol/L)] × [fasting insulin (mIU/L)]/22.5. GDM was defined as having one or more abnormal values from the 2-h 75-g OGTT between 24 and 28 weeks of gestation, with cutoff values of 5.1 mmol/L for fasting, 10.0 mmol/L for 1 h, and 8.5 mmol/L for 2 h [33]. Gestational weight gain was calculated as the body weight at delivery minus the body weight at baseline.

Placentas were collected immediately after delivery by midwives from the medical institution according to standard protocols. Briefly, biopsies of six points of the inner region of the placenta around the umbilical cord (the circumference of the radius was 4 cm) were obtained, rinsed by normal saline, immersed into a tube with RNAlater™ stabilization solution, and preserved at − 20 °C. Within a week, the samples were moved to a − 80 °C freezer.

### Genome-wide DNA differential methylation analysis and validation

Firstly, the genome-wide differential methylation analysis was used to identify significant DMRs, CpG sites, and genes (step 1). Secondly, we performed GO term enrichment analysis to identify CpG sites in genes that are related to insulin regulation (step 2). Thirdly, we selected CpG sites for validation and correlation analysis with maternal phenotypes in the regulatory regions of the selected insulin-related genes since these CpG sites are more likely to be functional to impact gene expression (step 3).

Genomic DNA was isolated from ground-up placentas using the QIAamp DNA Mini Kit (QIAGEN, Germany). Bisulfite conversion using the EZ DNA Methylation Kit (Zymo Research, Irvine, CA, USA) was carried out according to the manufacturer’s protocols. Genome-wide DNA methylation levels of 24 subjects were examined by Infinium 450K Human Methylation BeadChip (Illumina, San Diego, CA, USA), following the standard manufacturer’s manual. DNA from the two groups of subjects were randomized and assigned to two chips (12 samples on each chip) in a 1:1 ratio and processed by the same technician at the same time to minimize batch and chip effects of the two compared groups. On each chip, 12 DNA samples were randomly assigned to cells.

Signals of all 485,577 CpGs were analysed to distinguish from background with detection *P* values less than 0.01, according to the MinifiR package [34], which also estimated the methylation *β* value which is interpreted as the proportion of DNA molecules in the sample with methylation at a given site and ranged between 0 (unmethylated) and 1 (fully methylated). Probes with a bead count less than 3 in 5% or more of the samples, probes on chromosome X or Y, and the 65 control probes were also excluded. Finally, 434,958 CpG sites were available for differential analysis. The beta mixture quantile dilation (BMIQ) method was used for normalization [35]. Probe Lasso [36] using the ChAMP R package (version 1.8.2, http://bioconductor.org/packages/ChAMP/) was employed to identify differentially methylated regions (DMRs), and FDR-adjusted DMR *P* values were determined using a method by Benjamini and Hochberg [37]. Gene oncology (GO) terms and protein interaction (KEGG) enrichment analyses were performed for all CpG sites involved in significant DMRs (adjusted *P* < 0.05).

From 365 differentially methylated sites indicated by stage 1 analysis, those in the regulatory regions (promoter, 5′ UTR, and 3′ UTR) of insulin regulation genes and indicated by GO term analysis were selected for validation analysis. Literature research was undertaken to check the functions of these genes. Ten CpG sites in the promoter region or in the 3′ UTR region of four insulin resistance-related genes were selected for further examination and methylation–phenotype association analysis. For quality control, CpG sites in the regulatory region (3′ UTR or 5′ UTR) of the selected genes were validated using pyrosequencing in the discovery cohort (*n* = 24 subjects) followed by further analysis using all subjects with available placenta tissue samples (*n* = 153 subjects).

### mRNA expression analysis of the *SSTR4* gene

Total RNA was isolated from 50 to 100 mg placental tissue (stored at − 80 °C) using the Qiagen RNA mini kit, according to the manual’s instructions. Complementary DNA (cDNA) was synthesized using TAKARA reverse transcription kits (RR036A Takara PrimeScript™ RT Master Mix, Japan). The primers used were designed using Primer Premier 5.0 software. Primer sequences were as follows: *SSTR4* (forward) 5′-CCTTCGCTACGCCAAGATGA-3′ and (reverse) 5′-GAGACAGAAGACGCTGGTGAA-3′, yielding a PCR product length of 196 bp. GAPDH primer was (forward) 5′-GAAGGTCGGAGTCAACGGATT-3′ and (reverse) 5′-TGCTGATGATCTTGAGGCTGTT-3′, yielding a PCR product length of 434 bp. Total RNA mixed with RNase-free water (8 μL) and 2 μL PrimeScript RT Master Mix was added to a tube placed in an ice bath, and the contents settled to the bottom of the tube by spinning briefly for 3–5 s. The tube was placed in a 37 °C water bath (ABI PCR 2720) for 15 min and an 85 °C water bath for 5 s and then cooled on ice. cDNA was stored at − 20 °C until use.

RT-PCR was performed in triplicate to determine the *SSTR4* mRNA expression of the placental tissue in 153 subjects using the LightCycler 480 II (Roche). For amplification by PCR, 6 μL RNase-free water, 10 μL real-time PCR mix, 2 μL cDNA, 1 μL forward primer (10 μM), and reverse primer (10 μM) were added. Using GAPDH as an internal control, the amplification of the *SSTR4* gene was performed in the same tube under the following thermal cycling conditions: initial denaturation at 95 °C for 1 min, followed by 45 cycles of denaturation at 95 °C for 30 s, annealing at 60 °C for 30 s and extension at 72 °C for 30 s, and the last extension at 72 °C for 30 s. The melting curve included 1 cycle at 95 °C for 5 s and 65 °C for 1 min. The expression of *SSTR4* mRNA was estimated by the 2^-ΔΔCT^ method using G038 as controls.

### Placenta DNA methylation–maternal phenotype association analysis

Continuous phenotypes are described by their mean ± SD or median (interquartile range) where necessary, and categorical data are described by percentages. Comparisons of the characteristics of pregnant women between the control and the decreased GI group were performed by using independent sample *t* tests for continuous variables and chi-squared tests for categorical variables. Correlations between methylation levels, gene expression levels, and maternal phenotypes were analysed using a pairwise Spearman correlation analysis. Maternal phenotypes included changes of dietary GI, GL, fibre, carbohydrates, and serum insulin, as well as body weight gain across gestation. Contributions of the GI diet change to CpG site methylation levels were evaluated by explained *R*^2^ of stepwise multiple regression to adjust possible covariates. *P* < 0.05 was chosen as the significant level.

The protocol of the study was approved by the institutional ethics committee. Written informed consent forms were obtained from each subject at recruitment before any data collection.

## Additional files

Additional file 1: Significant DMRs and CpG sites based on the genome-wide differential methylation analysis (FDR-adjusted *P* < 0.05) and annotation information. (XLSX 148 kb)

Additional file 2: Significant GO terms by enrichment analysis based on CpG sites from four selective genes related to insulin regulation. (XLS 4 kb)

## Abbreviations

BMI: Body mass index
BMIQ: Beta mixture quantile dilation
DMR: Differentially methylated region
GDM: Gestational diabetes mellitus
GI: Glycaemic index
GL: Glycaemic load
GO: Gene ontology
OGTT: Oral glucose tolerance test
TSS: Transcription starting site
